# Supplementary material for: Inhibition of IRGM establishes a robust antiviral immune state to restrict pathogenic viruses
Source: EMBO Rep. 2021 Sep 1;22(11):e52948. doi: 10.15252/embr.202152948 (PMC8567234; doi:10.15252/embr.202152948)
Supplement: Supplementary file 1 — Expanded View Figures PDF [file EMBR-22-e52948-s006.pdf]

Expanded View Figures

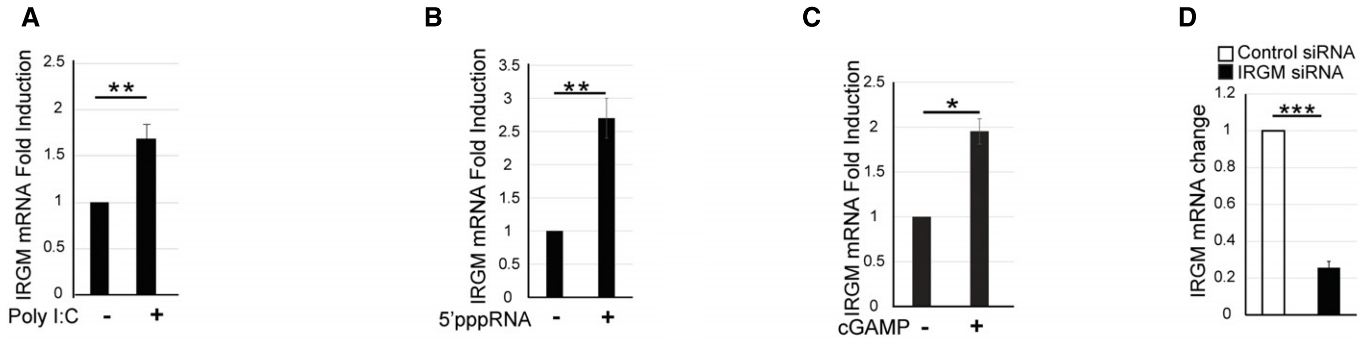

Figure EV1. Viruses and viral PAMP-induced IRGM expression suppress the interferon response.

A THP-1 cells were untreated or treated with Poly I:C (1 μg/ml) for 4 h and were subjected to qRT–PCR with IRGM (n = 3, mean ± SD, $^{**}P \le 0.005$ Student's unpaired t-test).

B THP-1 cells untreated or treated with 5'ppp-dsRNA (1 μg/ml) for 4 h and was subjected to qRT–PCR with IRGM (n = 3, mean ± SD, $^{**}P \le 0.005$, Student's unpaired t-test).

C THP-1 cells untreated or treated with cGAMP (1 μg/ml) for 4 h and was subjected to qRT–PCR with IRGM (n = 3, mean ± SD, $^{*}P < 0.05$, Student's unpaired t-test).

D The graph depicts the knockdown efficiency upon transfection of control and IRGM siRNA in THP-1 cells (n = 4, mean ± SD, $^{***}P < 0.0005$, Student's unpaired t-test).

 © 2021 The Authors

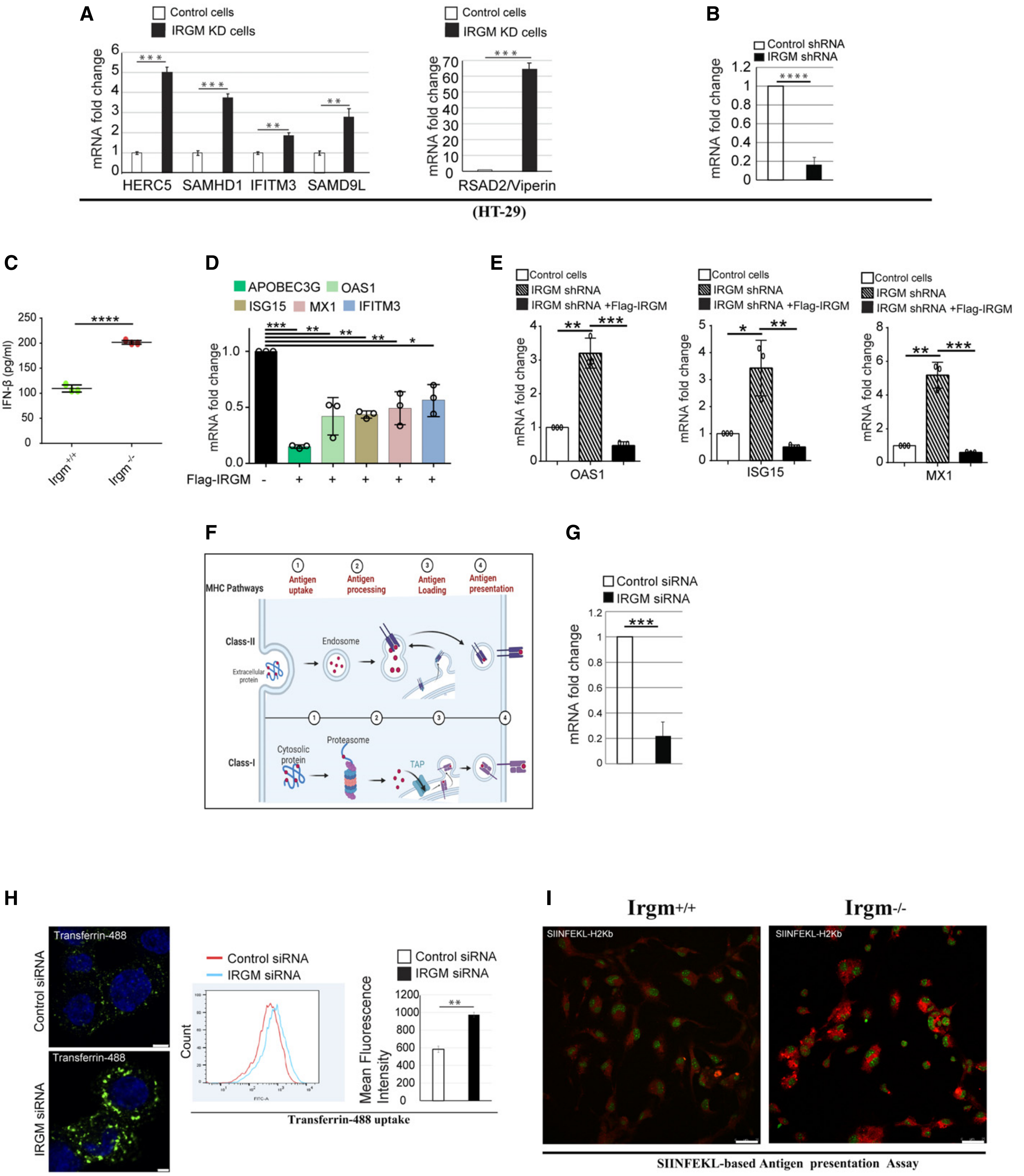
A
Control cells
IRGM KD cells
mRNA fold change
HERC5
SAMHD1
IFITM3
SAMD9L
RSAD2/Viperin
(HT-29)
B
Control shRNA
IRGM shRNA
C
IFN-β (pg/ml)
Irgm+/+
Irgm-/-
D
APOBEC3G
OAS1
ISG15
MX1
IFITM3
Flag-IRGM
E
Control cells
IRGM shRNA
IRGM shRNA +Flag-IRGM
OAS1
ISG15
MX1
F
MHC Pathways
Antigen uptake
Antigen processing
Antigen Loading
Antigen presentation
Class-II
Endosome
Extracellular protein
Class-I
Cytosolic protein
Proteasome
TAP
G
Control siRNA
IRGM siRNA
H
Transferrin-488
Control siRNA
IRGM siRNA
Count
Mean Fluorescence Intensity
Transferrin-488 uptake
I
Irgm+/+
Irgm-/-
SIINFEKL-H2Kb
SIINFEKL-based Antigen presentation Assay

Figure EV2.

Figure EV2. Key antiviral mechanisms are upregulated upon IRGM depletion.

A RNA isolated from control and IRGM knockdown HT-29 cells and subjected to qRT–PCR with indicated viral restriction factor genes (n = 3, mean ± SD, **P < 0.005, ***P < 0.0005, Student's unpaired t-test).
B The graph depicts the knockdown efficiency of control and IRGM shRNA stable HT-29 cells (n = 3, mean ± SD, ****P < 0.00005, Student's unpaired t-test).
C The graph depicts the IFN-β levels in serum of Irgm1 wild-type and KO mice (n = 3 mice each group, mean ± SD, ****P < 0.00005, Student's unpaired t-test).
D The qRT–PCR analysis of APOBEC3G, OAS1, ISG15, MX1 and IFITM3 with RNA isolated from control or Flag IRGM overexpressing HT-29 cells (n = 3, Mean ± SD, *P < 0.05, **P < 0.005, ***P < 0.0005, Student's unpaired t-test).
E The qRT–PCR analysis of OAS1, ISG15 and MX1 with RNA isolated from control or IRGM shRNA or Flag IRGM complemented IRGM shRNA HT-29 cells. n = 3, Mean ± SD, *P < 0.05, **P < 0.005, ***P < 0.0005, Student's unpaired t-test.
F Pictorial representation of sequential events of antigen uptake, processing, and presentation via Class I and Class II MHC Pathways. Created using Biorender.com.
G The graph depicts the knockdown efficiency upon transfection of control and si-IRGM in THP-1 cells (n = 3, mean ± SD, ***P < 0.0005, Student's unpaired t-test).
H Transferrin uptake assay shown by representative confocal images and flow cytometry analysis of control and si-IRGM transfected THP-1 cells treated with AF488 Transferrin (green) (10 μg/ml, 30 min). Graph depicts the mean fluorescence intensity of transferrin uptake in control and si-IRGM transfected THP-1 cells treated with AF488 transferrin. Scale, 5 μm (upper panel); Scale, 3 μm, (lower panel).
I Representative confocal images of H-2Kb-SIINFEKL (red) in Irgm1$^{+/+}$ and Irgm1$^{-/-}$ BMDMs treated with OVA (2 mg/ml, 3 h). Scale, 25 μm.

Figure EV3. IRGM-depleted cells are resistant to viral infection.

A Total RNA was isolated from mock HT-29 cells or CHIKV (MOI 1, 24 h) infected HT-29 cells or Flag IRGM overexpressing HT-29 cells and subjected to qRT–PCR with VSV specific primers to quantitate total viral load (n = 3, Mean ± SD, *P < 0.05, Student's unpaired t-test).
B Total RNA was isolated from mock and VSV (MOI 2.5, 24 h) infected control and IRGM knockdown HeLa cells and subjected to qRT–PCR with VSV specific primers to quantitate total viral load (n = 3, mean ± SE, ***P < 0.0005, Student's unpaired t-test).
C Left panels, representative fluorescence microscopic images of control and IRGM knockdown HeLa (MOI 2.5) and THP-1 (MOI 5) cells infected with VSV-eGFP for 4 h. Right panel, the graph depicts quantitative analysis of percentage of cells that are VSV-eGFP positive (n = 3, mean ± SD, **P < 0.005, ***P < 0.0005 Student's unpaired t-test).
D Left panels, representative fluorescence microscopic images of control or IRGM KD or Flag IRGM complemented IRGM KD THP-1 cells infected with VSV-eGFP (MOI 5.0) for 4 h. Scale, 400 μm. Right panel, the graph depicts VSV-eGFP viral mRNA fold change. n = 3, Mean ± SD, *P < 0.05, Student's unpaired t-test.
E Meta-analysis using Metascape, comparing the transcriptome significantly induced in Irgm1$^{-/-}$ mice (> 1.5 folds; P < 0.05) and the genes induced significantly upon SARS-CoV-2 infection (seven different conditions from four different studies). The gene expression from different conditions and studies cluster together along with the query gene list (marked with red).
F Circos plot depicting the gene overlap analysis between the genes induced in Irgm1$^{-/-}$ mice (> 1.5 folds; P < 0.05) and the genes induced significantly upon SARS-CoV-2 infection (seven different conditions from four different studies). On the outside, each arc represents the identity of each gene list. On the inside, each arc represents a gene list, where each gene has a spot on the arc. Dark orange color represents the genes that appear in multiple lists and light orange color represents genes that are unique to that gene list. Purple lines link the same gene that is shared by multiple gene lists. Blue lines link the different genes where they fall into the same ontology term (the term has to be statistically significantly enriched and with a size no larger than 100). The greater the number of purple links and the longer the dark orange arcs imply greater overlap among the input gene lists. Blue links indicate the amount of functional overlap among the input gene list
G The heatmap depicts statistically enriched terms (GO or KEGG or Reactome) obtained when induced genes in Irgm1$^{-/-}$ mice (> 1.5 folds; P < 0.05) were compared with the genes induced upon SARS-CoV-2 infection (seven different conditions from four different studies). The heatmap cells are colored by their P-values (P-values are calculated based on the accumulative hypergeometric distribution, and q-values are calculated using the Benjamini-Hochberg procedure to account for multiple testings as described at https://metascape.org/COVID/), white cells indicate the lack of enrichment for that term in the corresponding gene list.
H Amplification plot of qRT–PCR analysis conducted with SARS-CoV-2 nucleocapsid primers and RNA isolated from the supernatant of THP-1 cells infected with SARS-CoV-2 for 10 h and 20 h. The horizontal line (with values in red font) indicates threshold value of qPCR.
I Representative images of the plaque assay in Vero E6 cells performed from the culture supernatant of uninfected and SARS-CoV-2 (MOI 1, 24 h) infected THP-1 cells.
J Western blot analysis with cell lysates of control and si-IRGM transfected THP-1 and HT-29 cells and probed with the indicated antibodies.
K qRT–PCR analysis showing the percentage of viral infection in control or IRGM siRNA transfected THP-1 cells untreated or treated with N-acetyl-l-cysteine (NAC, 1 mM, 2 h) followed by VSV-eGFP infection (MOI 5.0) for 4 h. Mean ± SD, n = 3 (biological replicates), *P < 0.05, Student's unpaired t-test.

Source data are available online for this figure.

 © 2021 The Authors

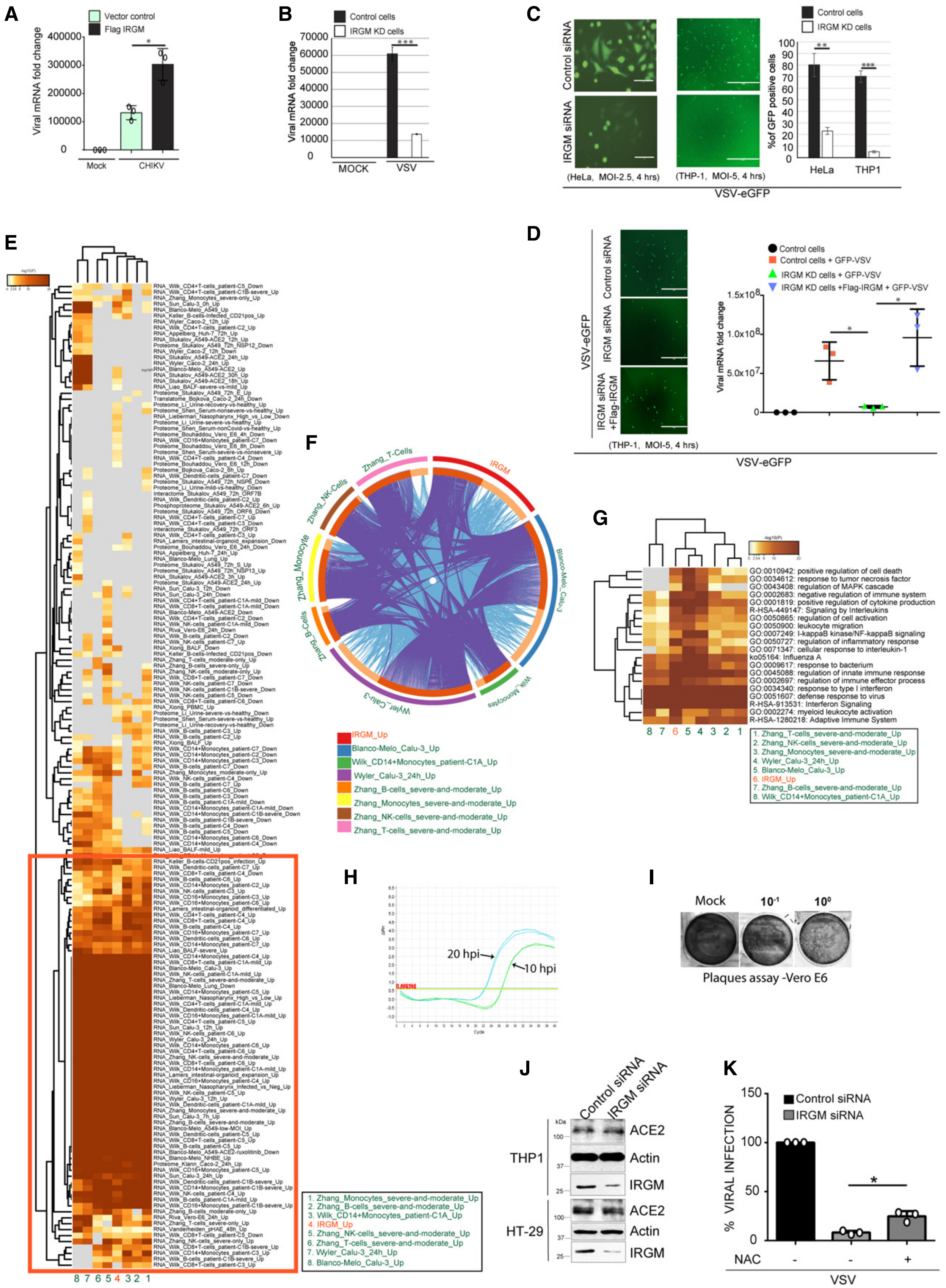
A
Vector control
Flag IRGM
Viral mRNA fold change
400000
300000
200000
100000
0
Mock
CHIKV
B
Control cells
IRGM KD cells
Viral mRNA fold change
70000
60000
50000
40000
30000
20000
10000
0
MOCK
VSV
C
Control siRNA
IRGM siRNA
(HeLa, MOI-2.5, 4 hrs)
(THP-1, MOI-5, 4 hrs)
Control cells
IRGM KD cells
%of GFP positive cells
HeLa
THP1
VSV-eGFP
D
VSV-eGFP
Control siRNA
IRGM siRNA
IRGM siRNA +Flag-IRGM
(THP-1, MOI-5, 4 hrs)
Control cells
Control cells + GFP-VSV
IRGM KD cells + GFP-VSV
IRGM KD cells +Flag-IRGM + GFP-VSV
Viral mRNA fold change
VSV-eGFP
E
8 7 6 5 4 3 2 1
1. Zhang_Monocytes_severe-and-moderate_Up
2. Zhang_B-cells_severe-and-moderate_Up
3. Wilk_CD14+Monocytes_patient-C1A_Up
4. IRGM_Up
5. Zhang_NK-cells_severe-and-moderate_Up
6. Zhang_T-cells_severe-and-moderate_Up
7. Wyler_Calu-3_24h_Up
8. Blanco-Melo_Calu-3_Up
F
IRGM
Blanco-Melo_Calu-3
Wilk_Monocytes
Wyler_Calu-3
Zhang_B-Cells
Zhang_Monocyte
Zhang_NK-Cells
Zhang_T-Cells
IRGM_Up
Blanco-Melo_Calu-3_Up
Wilk_CD14+Monocytes_patient-C1A_Up
Wyler_Calu-3_24h_Up
Zhang_B-cells_severe-and-moderate_Up
Zhang_Monocytes_severe-and-moderate_Up
Zhang_NK-cells_severe-and-moderate_Up
Zhang_T-cells_severe-and-moderate_Up
G
GO:0010942: positive regulation of cell death
GO:0034612: response to tumor necrosis factor
GO:0043408: regulation of MAPK cascade
GO:0002683: negative regulation of immune system
GO:0001819: positive regulation of cytokine production
R-HSA-449147: Signaling by Interleukins
GO:0050865: regulation of cell activation
GO:0050900: leukocyte migration
GO:0007249: I-kappaB kinase/NF-kappaB signaling
GO:0050727: regulation of inflammatory response
GO:0071347: cellular response to interleukin-1
ko05164: Influenza A
GO:0009617: response to bacterium
GO:0045088: regulation of innate immune response
GO:0002697: regulation of immune effector process
GO:0034340: response to type I interferon
GO:0051607: defense response to virus
R-HSA-913531: Interferon Signaling
GO:0002274: myeloid leukocyte activation
R-HSA-1280218: Adaptive Immune System
8 7 6 5 4 3 2 1
1. Zhang_T-cells_severe-and-moderate_Up
2. Zhang_NK-cells_severe-and-moderate_Up
3. Zhang_Monocytes_severe-and-moderate_Up
4. Wyler_Calu-3_24h_Up
5. Blanco-Melo_Calu-3_Up
6. IRGM_Up
7. Zhang_B-cells_severe-and-moderate_Up
8. Wilk_CD14+Monocytes_patient-C1A_Up
H
20 hpi
10 hpi
I
Mock
10⁻¹
10⁰
Plaques assay -Vero E6
J
Control siRNA
IRGM siRNA
THP1
HT-29
ACE2
Actin
IRGM
K
Control siRNA
IRGM siRNA
% VIRAL INFECTION
150
100
50
0
NAC
VSV

Figure EV3.
