# Supplementary material for: Inhibition of IRGM establishes a robust antiviral immune state to restrict pathogenic viruses
Source: EMBO Rep. 2021 Sep 1;22(11):e52948. doi: 10.15252/embr.202152948 (PMC8567234; doi:10.15252/embr.202152948)
Supplement: Supplementary file 2 — Movie EV1 [file EMBR-22-e52948-s009.zip › MovieEV1/MovieEV1_Legend.docx]

**Movie EV1.** The CHIKV infected *Irgm^+/+^* neonate mice at different stages of the disease.
