# Supplementary material for: Inhibition of IRGM establishes a robust antiviral immune state to restrict pathogenic viruses
Source: EMBO Rep. 2021 Sep 1;22(11):e52948. doi: 10.15252/embr.202152948 (PMC8567234; doi:10.15252/embr.202152948)
Supplement: Supplementary file 3 — Movie EV2 [file EMBR-22-e52948-s002.zip › MovieEV2/MovieEV2_Legend.docx]

**Movie EV2.** The CHIKV infected *Irgm^+/+^* neonate mice before the leg paralysis was observed.
