# Supplementary material for: Inhibition of IRGM establishes a robust antiviral immune state to restrict pathogenic viruses
Source: EMBO Rep. 2021 Sep 1;22(11):e52948. doi: 10.15252/embr.202152948 (PMC8567234; doi:10.15252/embr.202152948)
Supplement: Supplementary file 4 — Movie EV3 [file EMBR-22-e52948-s008.zip › MovieEV3/MovieEV3_Legend.docx]

**Movie EV3.** The CHIKV infected *Irgm^+/+^* and *Irgm^-/-^* mice. The *Irgm^+/+^* mice are at the single leg paralysis stage.
