# Supplementary figures and images for: Inhibition of IRGM establishes a robust antiviral immune state to restrict pathogenic viruses
Source: EMBO Rep. 2021 Sep 1;22(11):e52948. doi: 10.15252/embr.202152948 (PMC8567234; doi:10.15252/embr.202152948)

Figure-EV3

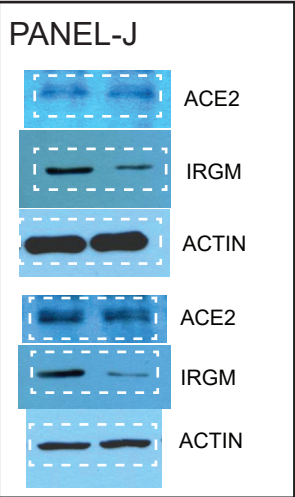

ACTIN

Supplement: Supplementary file 6 — Source Data for Figure EV3 [file EMBR-22-e52948-s003.pdf]

Figure-1

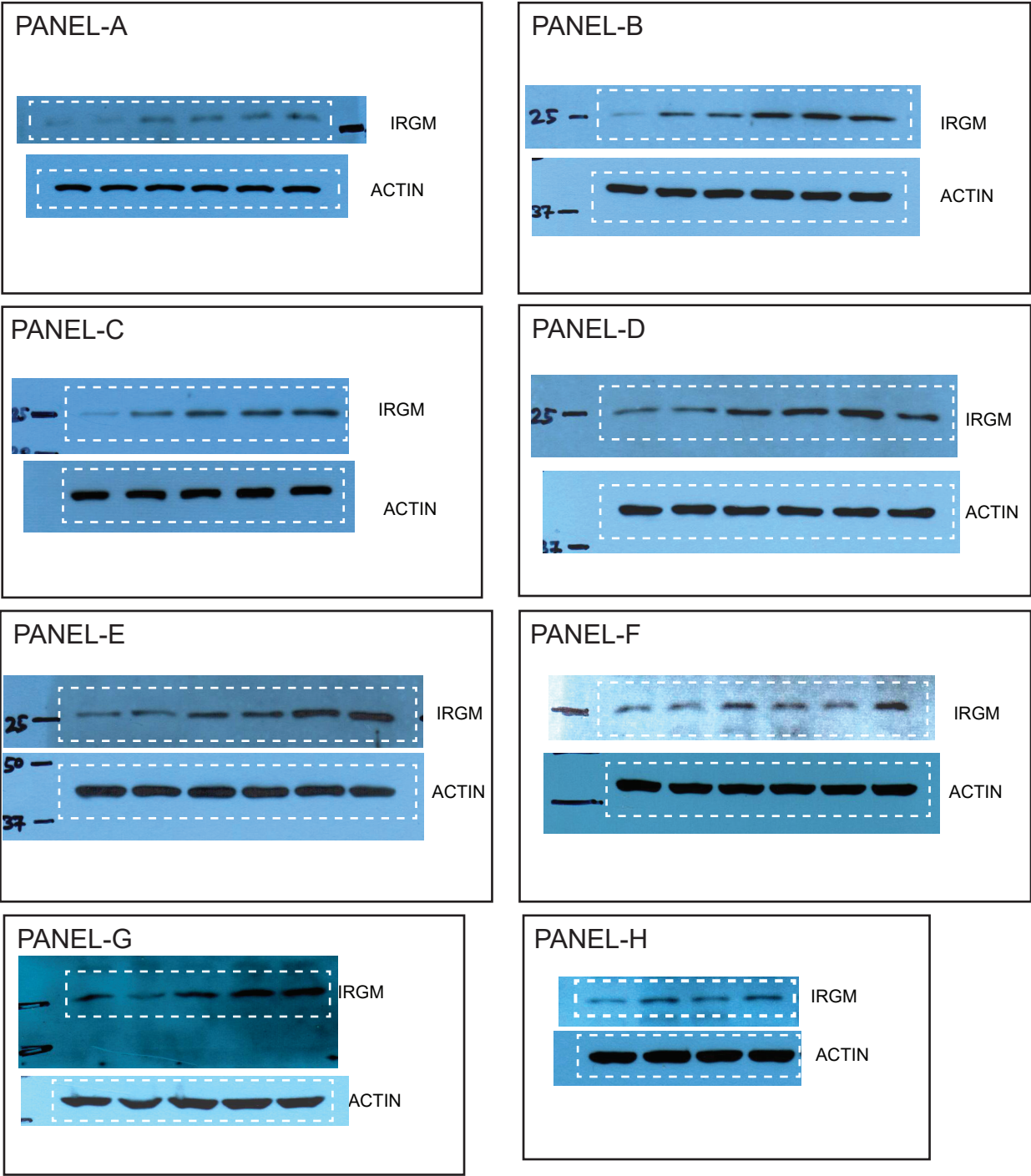

Supplement: Supplementary file 7 — Source Data for Figure 1 [file EMBR-22-e52948-s007.pdf]

Figure-2

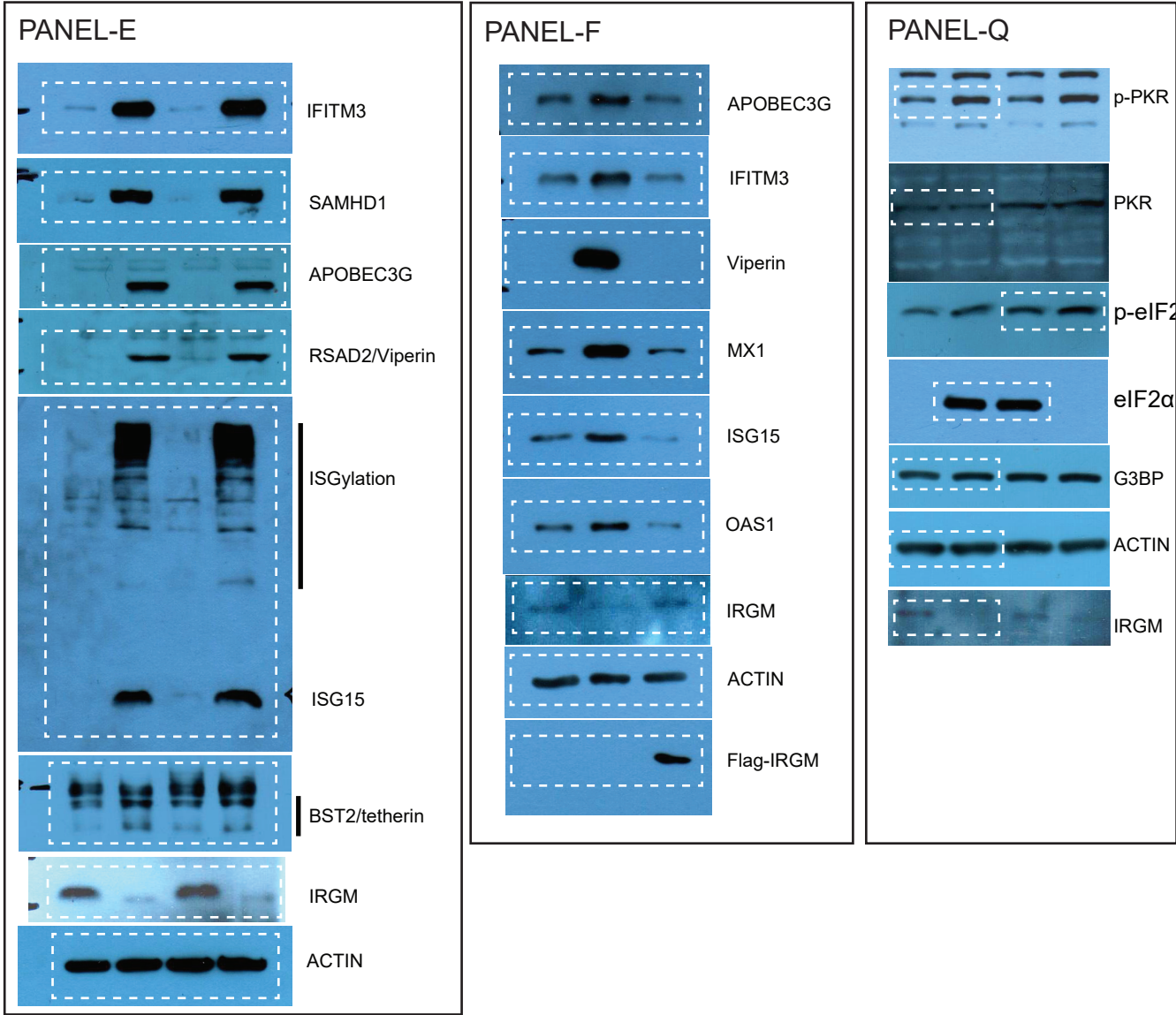

Supplement: Supplementary file 8 — Source Data for Figure 2 [file EMBR-22-e52948-s004.pdf]

Figure-5

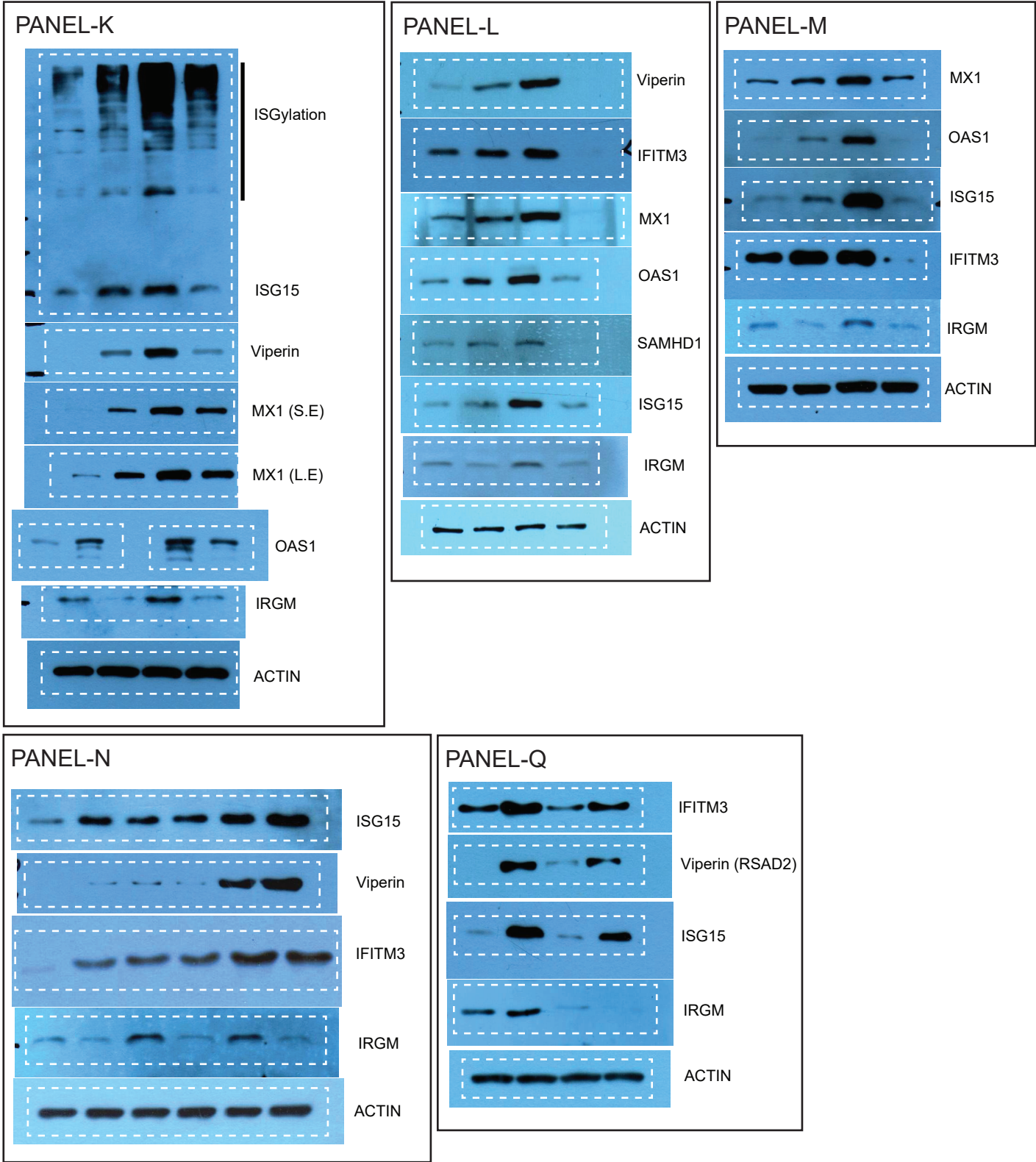

Supplement: Supplementary file 9 — Source Data for Figure 5 [file EMBR-22-e52948-s005.pdf]
